# Metabolic Engineering of *Saccharomyces cerevisiae* for Enhanced Carotenoid Production From Xylose-Glucose Mixtures

**DOI:** 10.3389/fbioe.2020.00435

**Published:** 2020-05-14

**Authors:** Buli Su, Dandan Song, Honghui Zhu

**Affiliations:** State Key Laboratory of Applied Microbiology Southern China, Guangdong Provincial Key Laboratory of Microbial Culture Collection and Application, Guangdong Microbial Culture Collection Center (GDMCC), Guangdong Institute of Microbiology, Guangdong Academy of Sciences, Guangzhou, China

**Keywords:** *Saccharomyces cerevisiae*, xylose-glucose mixtures, Pho13, Gal2, carotenoid

## Abstract

Co-utilization of xylose and glucose from lignocellulosic biomass is an economically feasible bioprocess for chemical production. Many strategies have been implemented for efficiently assimilating xylose which is one of the predominant sugars of lignocellulosic biomass. However, there were few reports about engineering *Saccharomyces cerevisiae* for carotenoid production from xylose-glucose mixtures. Herein, we developed a platform for facilitating carotenoid production in *S. cerevisiae* by fermentation of xylose-glucose mixtures. Firstly, a xylose assimilation pathway with mutant xylose reductase (XYL1m), xylitol dehydrogenase (XYL2), and xylulokinase (XK) was constructed for utilizing xylose. Then, introduction of phosphoketolase (PK) pathway, deletion of *Pho13* and engineering yeast hexose transporter Gal2 were conducted to improve carotenoid yields. The final strain SC105 produced a 1.6-fold higher production from mixed sugars than that from glucose in flask culture. In fed-batch fermentation with continuous feeding of mixed sugars, carotenoid production represented a 2.6-fold higher. To the best of our knowledge, this is the first report that *S. cerevisiae* was engineered to utilize xylose-glucose mixtures for carotenoid production with a considerable high yield. The present study exhibits a promising advantage of xylose-glucose mixtures assimilating strain as an industrial carotenoid producer from lignocellulosic biomass.

## Introduction

Glucose from starch hydrolysates was widely used as raw material in industrial biotechnology (Buschke et al., 2013). In order to avoid competing directly with human nutrition derived from limited land resources, lignocellulose biomass containing the main building blocks of glucose and xylose was regarded as the most promising alternative raw material (Jagtap and Rao, 2018). Xylose is the second abundant sugar from lignocellulose which can account for 30–40% of lignocellulosic biomass (Kwak and Jin, 2017). Therefore, it is important to utilize both glucose and xylose from lignocellulosic biomass during manufacturing process for the economically feasible production of valuable chemicals.

Recent years, the microbial transformations of xylose into various chemicals, including xylitol, ethanol, butanol, squalene, 2,3-butanediol and lipid, were extensively studied (Kwak et al., 2019). However, the difficulty of engineering an efficient microbial cell factory for utilizing xylose hindered fully using lignocellulosic biomass (Zhang et al., 2019). Not only naturally xylose-assimilating yeasts, but also non-xylose assimilating yeasts have been developed as a platform for biosynthesis of biofuels and chemicals from lignocellulosic biomass. Especially, non-xylose-assimilating *S. cerevisiae* has been extensively studied for bioethanol production from xylose in lignocellulosic biomass due to its remarkable alcoholic fermentation (Kwak et al., 2019). Since *S. cerevisiae* lacked the capability of xylose assimilation, strategies such as engineering endogenous pathway were not viable. Instead, metabolic engineering strategies including introduction of heterogenous *XYL1/XYL2* for xylose metabolism, overexpression of xylose isomerase or *XK* from various sources to obtain a balanced metabolic pathway, improvement of the transport efficiency through engineering heterogeneous or native xylose transporters, have been implemented to facilitate xylose utilization (Kim et al., 2013a). Nevertheless, strains widely used in industry lacked the ability to convert xylose into valuable products, and only few reports were available on making investigation of fermentation for isoprenoid production from xylose-glucose mixtures (Gao et al., 2018). For example, *S. cerevisiae* was firstly demonstrated to synthetize zeaxanthin from glucose or xylose (or xylan) (Sun et al., 2012).

**FIGURE 1:**
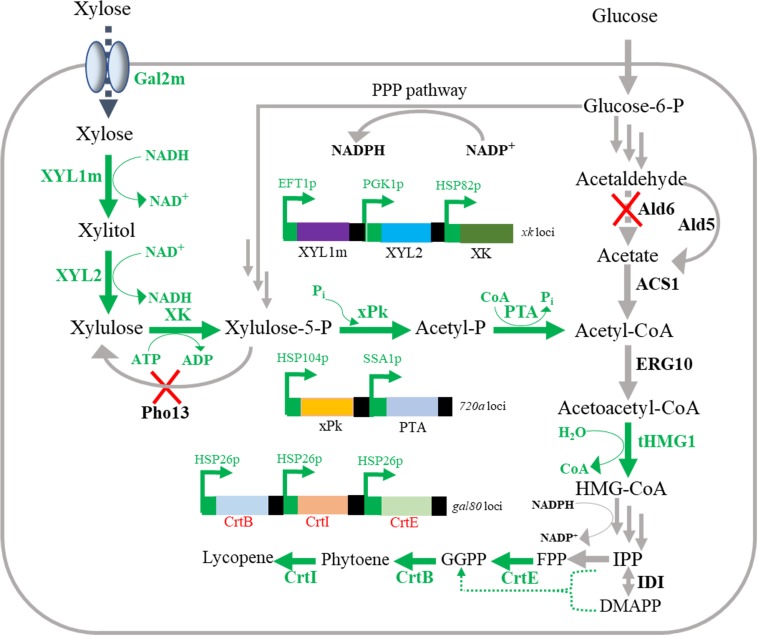
Overview of lycopene production from xylose-glucose mixtures in engineered *Saccharomyces cerevisiae*. Green words represent the heterogeneous expression pathways; blue words represent the native pathways; and red forks represent genes deletion in this study. XYL1m, mutant xylose reductase; XYL2, xylitol dehydrogenase; XK, xylulokinase; Pho13, para-nitrophenyl phosphatase; xPk, xylulose-5-phosphate specific phosphoketolase; PTA, phosphotransacetylase; Ald6, Ald5, aldehyde dehydrogenase; ACS1, acetyl-CoA synthetase; ERG10, acetyl-CoA acetyltransferase; tHMG1, truncated hydroxymethylglutaryl-CoA reductase; IDI, isopentenyl-diphosphate isomerase; CrtE, geranylgeranyl diphosphate synthase; CrtB, phytoene synthase; CrtI, phytoene desaturase; HMG-CoA, hydroxymethylglutaryl-CoA; IPP, isopentenyl diphosphate; DMAPP, dimethylallyl diphosphate; FPP, farnesyl diphosphate; GGPP, geranylgeranyl diphosphate.

The aim of the present study is to develop a *S. cerevisiae* platform that can utilize mixed sugars for carotenoid production (Figure 1). To illustrate improved carotenoid production from xylose-glucose mixtures, lycopene was chosen as a model carotenoid. We selected a basic lycopene-producing *S. cerevisiae* strain, namely BL03-D-4 (unpublished), as our starting strain. Firstly, XYL1m (NADH-dependent), and XYL2 were introduced into BL03-D-4 to utilize xylose. Then, introduction of PK pathway, deletion of *Pho13* and engineering yeast hexose transporter Gal2 were applied. Consequently, a 2.6-fold higher production was obtained from xylose-glucose mixtures than that from glucose in the continuous fermentation. To the best of our knowledge, this is the first report that *S. cerevisiae* was engineered to utilize xylose-glucose mixtures for carotenoid production with a considerable high yield. A shorter carotenoid synthetic route and an optimized mevalonic acid (MVA) pathway might account for the higher carotenoid production compared with previous work (Sun et al., 2012). From the comparison of the model carotenoid production from mixed sugars and glucose by engineered *S. cerevisiae*, we conclude that the assimilation of mixed sugars is a practicable approach for the overproduction of carotenoid in *S. cerevisiae* from lignocellulose-derived sugars.

## Materials and Methods

### Strains and Culture Conditions

Yeast strains and different plasmids used in this study are listed in Table 1. Primers are listed in Supplementary Table S1. *E. coli* DH5α was used for cloning and plasmid propagation. *E. coli* was grown at 37°C and 200 rpm in Luria-Bertani (LB) medium (5 g L^–1^ yeast extract, 10 g L^–1^ tryptone, and 10 g L^–1^ NaCl; 20 g L^–1^ agar was added for solid medium). *S. cerevisiae* was cultivated in YPD medium (10 g L^–1^ of yeast extract, 20 g L^–1^ of peptone and 20 g L^–1^ of glucose).

**TABLE 1:** Strains and plasmids used in this study.

**Strain/plasmid**	**Description**	**Sources**
**Strains**		
DH5α	*supE44 ΔlacU169* (φ*80 lacZ*Δ*M15*) *hsdR17 recA1 endA1 gyrA96 thi-1 relA1*	Invitrogen
*BY4742*	*MAT*α, *his3*Δ*1*, *leu2*Δ*0*, *lys2*Δ*0*, *ura3*Δ*0*	Su et al., 2020
BL03-D-4	*BY4742*, Δ*Gal80*:: *P_HSP__26_-CrtB-T_*ADH*__1_-P_HSP__26_-CrtI-T_*GPM*__1_-P_HSP__26_-CrtE-T_*CYC*__1_*,Δ*416d P_*Cit*__1_-tHMGR-T_*Guo*,_* Δ*Ald6*	Unpublished
SC101	BL03-D-4, Δ*Pxk*:: *P_EFT__1_-XYL1-T_*syn*__*th*__7_-P_PGK__1_-XYL2-T_guo__1_-P_HSP__82_*	This study
SC102	BL03-D-4, Δ*Pxk*:: *P_EFT__1_-XYL1m-T_*syn*__*th*__7_-P_PGK__1_-XYL2-T_guo__1_-P_HSP__82_*	This study
SC103	SC102,Δ*720a*:: *P_HSP__104_-xPk-T_guo__1_-P_SSA__1_-PTA-T_*syn*__*th*__6_*	This study
SC104	SC103,Δ*Pho13*	This study
SC105	SC104,Δ*P_*Gal*__2_*::*P_SSA__1_-Gal2m*	This study
SC106	SC105,Δ911b :: His3-Leu2-Ura3	This study
**Plasmids**		
pHCas9-gRNA	pBR322-derived vector, including HCas9, KanMX, gRNA and 2 μ ori, ampicillin resistant	Su et al., 2020
pHCas9-XK	pHCas9-gRNA targeted to the XK loci	This study
pHCas9-Pho13	pHCas9-gRNA targeted to the Pho13 loci	This study
pHCas9-Gal2	pHCas9-gRNA targeted to the Gal2 loci	This study
pHCas9-720a	pHCas9-gRNA targeted to the 720a loci	Su et al., 2020
pHCas9-911b	pHCas9-gRNA targeted to the 911b loci	Su et al., 2020
pACYC-XK	Plasmid including the modules of *P_EFT__1_-XYL1-T_*syn*__*th*__7_-P_PGK__1_-XYL2-T_guo__1_-P_HSP__82_*	This study
pACYC-PK	Plasmid including the modules of *P_*HSP104*_-xPk-T_*guo1*_-P_*SSA1*_-PTA-T_*synth6*_*	This study
pACYC-pro	Plasmid including the modules of His3-Leu2-Ura3	Su et al., 2020

### Strains Construction

For CRISPR-Cas9 mediated genome editing, the plasmid pHCas9M-gRNA constructed by our previous work (Molecular Cloud Cat. No.: MC_0000739) was used. BL03-D-4 (unpublished) contained overexpression cassettes for lycopene production, was used as the host for the development of carotenoid producing platform from mixed sugars. BL03-D-4 was constructed through overexpression of truncated form of 3-hydroxy-3-methylglutaryl-coenzyme-A reductase (tHMG1) and deletion of Ald6 in strain BL03 which contained codon optimized *crtE*, *crtB*, *crtI* genes (Su et al., 2020). The pHCas9M-gRNA plasmid contains a Cas9 nuclease expression module and a loci targeting gRNA expression module. For construction of loci specific plasmid, the specific 20 bp guide DNA sequence at the 5′-end of the gRNA targeting the appropriate region on chromosome was synthesized in primers. The target sequence within the appropriate locus was selected using the CRISPRdirect online tool (Naito et al., 2015). Cas9 nuclease expression module was amplified from pHCas9M-gRNA with primers gRNA-UP-F and gRNA-UP-R. Loci targeting gRNA expression modules for XK, Pho13 and Gal2 loci were amplified from pHCas9M-gRNA with primers gRNA-XK-F, gRNA-Pho13-F, gRNA-Gal2-P1, and gRNA-R, respectively. Then these two modules were connected according to the protocol of ClonExpress MultiS One Step Cloning Kit (Vazyme, China). The genome DNA of *S. cerevisiae* S288C was used as the template for amplifying promoters, and the synthetic terminators were used referring to previous literature (Curran et al., 2015). P_EFT__1_-XYL1-T_synth__7_-P_PGK__1_-XYL2-T_guo__1_-P_HSP__82_ module was constructed by overlapping PCR using primers EFT1-F-2, EFT1-F, EFT1-R (amplified P_EFT__1_ promoter with ∼50 bp left homologous arms), XYL1-F, XYL1-R, XYL1-R-2 (amplified XYL1 Gene ID: 4839234 from *Scheffersomyces stipitis* with terminator T_synth__7_), PGK-F, PGK-R (amplified P_PGK__1_ promoter), XYL2-F, XYL2-R, XYL2-R-2 (amplified XYL2 Gene ID: 4852013 from *S. stipitis* with terminator T_guo__1_) and HSP82-F, HSP82-R, HSP82-R-2 (amplified P_HSP__82_ promoter with ∼50 bp right homologous arms). P_HSP__104_-xPk-T_guo__1_-P_SSA__1_-PTA-T_synth__6_ module was also constructed by overlapping PCR using primers 720-F, HSP104-F, HSP104-R (amplified P_HSP__104_ promoter with ∼50 bp left homologous arms), xpk-F, xpk-R, xpk-R-2 (amplified xPk GenBank: TJY30451.1 from *Leuconostoc mesenteroides* with terminator T_guo__1_), SSA1-F0220, SSA1-R0220 (amplified P_SSA__1_ promoter) and pta-F, pta-R, pta-R-2, 720-R (amplified PTA GenBank: EEP53689.1 from *Clostridium butyricum* with terminator T_synth__6_ and ∼50 bp right homologous arms). Then, these expression modules were cloned into plasmid pACYC-Duet1. Pho13 deletion cassette was constructed by overlapping PCR using primers Pho13-UP-F, Pho13-UP-R (amplified ∼800 bp left homologous arms) and Pho13-DOWN-F, Pho13-DOWN-R (amplified ∼800 bp right homologous arms). For mutation of XYL1^K271N^, the P_EFT__1_-XYL1-T_synth__7_-P_PGK__1_-XYL2-T_guo__1_-P_HSP__82_ module was divided into two parts and the mutant point was synthesized in primers for construction of P_EFT__1_-XYL1m-T_synth__7_-P_PGK__1_-XYL2-T_guo__1_-P_HSP__82_ module using primers EFT1-XYL1-F, EFT1-XYL1-R, PGK-XYL2-F and PGK-XYL2-R. To obtain the Gal2^N376*F*^ mutant, P_SSA__1_-Gal2m module was constructed by overlapping PCR using primers SSA1-F0523-2, SSA1-F0523, SSA1-R (amplified P_SSA__1_ promoter with ∼50 bp left homologous arms) and Gal2-F, Gal2-R, Gal2-R-2 (amplified partial Gal2m with ∼50 bp right homologous arms). For transformation, the expression modules were amplified by PCR from the corresponding plasmids and were transformed into yeasts along with specific genome editing plasmid to accomplish the genetic modification. Yeast transformation was performed using the electroporation protocol (Wu and Letchworth, 2004). After genome editing, 400 μg mL^–1^ of G418 was added to the YPD plate for screening the correct transformants. Then, the correct transformants were further confirmed by diagnostic PCR using suitable primers (Supplementary Table S1) spanning both ends of the integrated construct.

### Yeast Culture for Carotenoid Production

For shake-flask fermentations, several colonies from a freshly agar plate were grown in 5 mL of YPD medium in a shaker (New Brunswick Scientific, NJ, United States) cultivated at 200 rpm and 30°C for overnight growth, then the seed broth was inoculated into fermentation broth with an initial optical density 0.5 (OD_600_). To obtain carotenoid production by different strains on glucose (or xylose) or mixed sugars, shake-flask fermentations were performed with 50 mL of modified YP medium containing 30 g L^–1^ xylose and 10 g L^–1^ glucose (or 40 g L^–1^ glucose or 40 g L^–1^ xylose) in 250 mL flasks at 30°C, 200 rpm.

For continuous fermentation, the seed broth was inoculated in 500 mL flask with 250 mL YPD medium and cultivated for 12–18 h at 30°C, 200 rpm. The fed-batch fermentation was conducted in 5 L fermenter (FUS-5, Guoqiang, Shanghai, China) with the YPD medium. Additional mixed sugars and medium were added to the bioreactor. Modified YP medium contained 10 g L^–1^ KH_2_PO_4_, 2.5 g L^–1^ MgSO_4_, 3.5g L^–1^ K_2_SO_4_, 0.25 g L^–1^ Na_2_SO_4_ and 1 mL trace metal solution (MgCl_2_⋅6H_2_O 250 mg L^–1^, CaCl_2_⋅2H_2_O 104.5 mg L^–1^, ZnSO_4_⋅7H_2_O 6.25 mg L^–1^, FeSO_4_⋅7H_2_0 3.5 mg L^–1^, CuSO_4_⋅5H_2_O 0.4 mg L^–1^, MnCl_2_⋅4H_2_O 0.1 mg L^–1^, Na_2_MoO_4_⋅2H_2_0 0.5 mg L^–1^, CoCl_2_⋅6H_2_O 0.3 mg L^–1^, H_3_BO_3_ 1 mg L^–1^, and KI 0.1 mg L^–1^).

### Quantitative Analysis

Total carotenoid was extracted according to the literature with some modifications (Xie et al., 2015). Briefly, cell cultures were washed after 96 h cultivation, resuspended in 3 mol L^–1^ HCl and boiling for 4 min, then cooled in baths of ice for 4 min. Next, cells debris were washed twice and resuspended in acetone vortexed followed by centrifugation. The acetone supernatant was transferred into a new tube for measuring total carotenoid. The total carotenoid production of different strains was determined by UV/Vis spectrometer (PerkinElmer Lambda 45) at 470 nm (Supplementary Figure S1). The extinction coefficient was adopted in acetone using an A 1% 1cm of 3450 (Scott, 2001).

The growth curve of different strains was determined by monitoring OD_600_ using a UV/Vis spectrometer. Dry cell weights (DCW) of different strains on glucose or mixed sugars were determined through drying the cells at 80°C for 24 h. The yields of carotenoids were expressed as mg per g dry cell weight (mg g^–1^ DCW) (Xie et al., 2015). Concentrations of glucose and xylose in culture broth were analyzed by Glucose assay Kit and D-Xylose assay kit according to the protocol, respectively (Nangjing Jiangcheng Bioengineering Institute, Nanjing, China). The secretory xylitol was analyzed using D-sorbitol/xylitol enzymatic test kits (Megazyme Assay Kits, Megazyme International Ireland Limited, Wicklow, Ireland). Acetic acid concentration was measured with an acetic acid assay kit [Acetic Acid (GK) Kit, Megazyme].

## Results

### Rational Engineering a Xylose-Assimilating Strain for Carotenoid Biosynthesis

**FIGURE 2:**
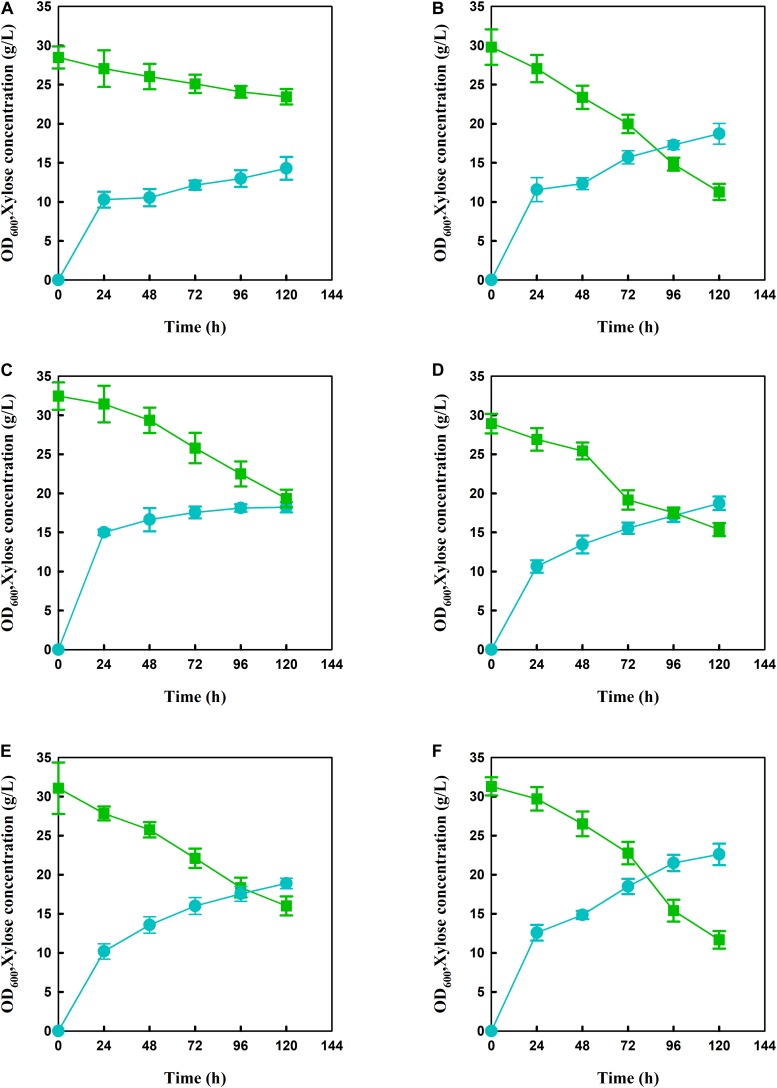
Growth curve and xylose consumption of engineered strains during xylose-glucose fermentation in flasks. **(A)** BL03-D-4, **(B)** SC101, **(C)** SC102, **(D)** SC103, **(E)** SC104, and **(F)** SC105 in modified YP medium with 10 g L^–1^ glucose and 30 g L^–1^ xylose for 120 h. Symbols: circles, OD_600_; triangles, xylose concentration. Since glucose was exhaust within 10 h, the glucose consumption was not presented in Figures. The error bars represent the average ± SD of three biological replicates.

In order to produce xylose-based carotenoid, the xylose-assimilating pathway including *XYL1/XYL2* was introduced into the lycopene-producing strain *S. cerevisiae* BL03-D-4 to use xylose as a carbon source, which was necessary for xylose-based production. Meanwhile, the endogenous *XK* was also overexpressed through replacing promoter *P*_*XK*_ with a relative strong promoter *SSA1* to obtain SC101 (Figure 1). Then, shake-flask fermentation was performed for strain SC101 in 50 mL modified YP broth with 30 g L^–1^ xylose and 10 g L^–1^ glucose. The growth curve and xylose consuming were detected throughout the cultivation. The results (Figure 2) demonstrated that SC101 could assimilate xylose and obtain a slight higher biomass than BL03-D-4 but far below than the previous work (Kwak et al., 2017). Approximately 18 g L^–1^ of xylose was consumed, and this result implied that a restrained metabolic flux through non-oxidative pentose phosphate pathway (PPP) existed in the present xylose-assimilating pathway (Gao et al., 2018). However, SC101 produced a 2.4-fold higher carotenoid than BL03-D-4 from mixed sugars. This result indicated that a xylose-assimilating pathway had a positive effect on carotenoid production.

**FIGURE 3:**
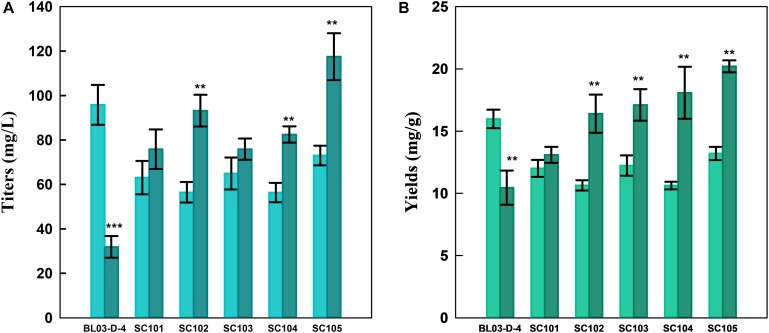
**(A)** Product titers and **(B)** yields of different strains from glucose (light color) or xylose-glucose mixtures (dark color) in flasks fermentation. Cultures were carried out in modified YP medium with 40 g L^–1^ glucose or 10 g L^–1^ glucose and 30 g L^–1^ xylose at 30°C for 96 h. Data represent the average ± SD of three biological replicates. Statistically significant differences are denoted **P < 0.01 and ***P < 0.001 (two-tailed Student’s *t*-test).

As shown in Figure 3, the xylose-based carotenoid titer of SC101was similar with the glucose-based titer. These results revealed that the xylose consuming might be a rate-limiting step for carotenoid synthesis (Katahira et al., 2017). Thus, a mutant *XYL1* (K271N, NADH preferred) (Bengtsson et al., 2009) was overexpressed to obtain SC102 for increasing xylose-based carotenoid production. This, in turn, more NAD might be available for the XDH reaction, and resulted in higher xylose assimilating rate. Although, we found that carotenoid yield could be increased in some extent (1.25-fold), the consumption of xylose was even slower than SC101 with the wild-type XYL1 (Table 2). This was due to the fact that xylitol which was derived from xylose was accumulated in the strain with the wild-type XYL1 (7.63 ± 0.95 g L^–1^) and more xylose flow into Embden-Meyerhof-Parnas (EMP) pathway in the strain with XYL1m (2.13 ± 0.61 g L^–1^).

**TABLE 2:** Fermentation parameters of different modified strains.

**Strains**	**Xylose consumed rate (g L^–1^ h^–1^)**	**Ycar/con (mg g^–1^)**	**Growth rates (h^–1^)**	**Acetic acid (g L^–1^)**	**Productivity (mg L^–1^ h^–1^)**	**Biomass (g L^–1^)**	**Final carotenoid yield (mg g DCW ^–1^)**
BL03-D-4	0.04 ± 0.004	2.05 ± 0.17	0.09 ± 0.004	0.53 ± 0.15	0.27 ± 0.04	3.04 ± 0.06	10.46 ± 1.38
SC101	0.15 ± 0.01	2.60 ± 0.14	0.10 ± 0.005	0.70 ± 0.20	0.63 ± 0.07	5.79 ± 0.53	13.10 ± 0.65
SC102	0.11 ± 0.02	3.87 ± 0.44	0.11 ± 0.001	0.77 ± 0.21	0.78 ± 0.06	5.74 ± 0.90	16.40 ± 1.53
SC103	0.11 ± 0.004	3.02 ± 0.11	0.10 ± 0.003	1.80 ± 0.66	0.63 ± 0.04	4.47 ± 0.63	17.11 ± 1.26
SC104	0.13 ± 0.03	3.28 ± 0.44	0.09 ± 0.004	1.27 ± 0.35	0.68 ± 0.03	4.62 ± 0.76	18.08 ± 2.08
SC105	0.16 ± 0.008	3.84 ± 0.58	0.11 ± 0.003	1.23 ± 0.40	0.98 ± 0.09	5.81 ± 0.39	20.20 ± 0.48

*Ycar/con, carotenoid yield (mg g**^–^**^1^ consumed sugars), DCW, Dry cell weights. Fermentations were performed for 120 h in YP medium supplemented with 30 g L**^–^**^1^ xylose and 10 g L**^–^**^1^ glucose.*

### Introduction of Phosphoketolase Pathway for Carotenoid Production

Acetyl-CoA is an important precursor for carotenoid synthesis and its biosynthesis was primary catalyzed by pyruvate decarboxylase (PDC), acetaldehyde dehydrogenase (Ald) and acetyl-CoA synthetase (ACS) in *S. cerevisiae* cytoplasm (van Rossum et al., 2016). Pyruvate was produced from glucose or xylose through EMP pathway, and then pyruvate was used as the precursor for acetyl-CoA synthesis via the PDH bypass (Kwak et al., 2019). There were many steps for converting glucose or xylose to acetyl-CoA. Therefore, a large proportion of xylose would be consumed for cell growth in the fermentation of xylose-glucose mixtures.

Alternatively, in this study, strain SC103 was constructed for improving carotenoid yield through introduction of PK pathway which included xylulose-5-phosphate phosphoketolase (xPK) and phosphotransacetylase (PTA) (Kocharin et al., 2013; Meadows et al., 2016). Xylose-5-phosphate was converted to acetyl-CoA through PK pathway which could greatly retrench the pathway for acetyl-CoA synthesis from xylose (Figure 1). In the fermentation of mixed sugars, xylose was ideally consumed for biosynthesis of target products, while glucose was used for growth and supply cofactor (Su et al., 2015). To achieve this, the native transaldolase (TAL1) and transketolase (TKL1) which could greatly improve xylose assimilation were not overexpressed in this study (Gao et al., 2018). As shown in Table 2, the xylose consumption shown no improvement in SC103 compared to SC102. Meanwhile, overexpression of PK pathway exerted slight inhibition on the growth of strain SC103. The decreased cell growth might ascribe to that the expression of PK pathway was coupled with an increased acetate production (Table 2; Bergman et al., 2019). The titers of carotenoid reached 64.9 and 75.9 mg L^–1^ from glucose and mixed sugars, respectively (Figure 3). Meanwhile, the yield of carotenoid from xylose-glucose was 17.1 mg g^–1^ DCW, which represented a 1.4-fold higher than that from glucose (12.2 mg g^–1^ DCW).

### Deletion of *Pho13* Exerts No Significant Effect on Xylose Consumption

In order to further improve xylose utilization, we assessed the effect of *Pho13* deletion on xylose assimilating pathway. Previous study has reported that *Pho13* deletion played an important role in improving xylose consumption rate and tolerance to toxic chemicals (Van Vleet et al., 2008). Herein, we deleted *Pho13* gene in strain SC103 described above to generate strain SC104. By deleting *Pho13*, as shown in Table 2, SC104 obtained slightly increase of the cell growth and xylose assimilation. We were surprised by this result, for that *XK* was also overexpressed using a strong promoter *SSA1* in SC104. This might due to that the facilitation of *Pho13* deletion was only effective in strains which harbored xylose-assimilating pathway with quite high overexpression of *XK* (Kim et al., 2013b). For that *Pho13* might cause a futile cycle by dephosphorylating xylulose-5-phosphate which would result in ATP waste and flux blockage along with highly active XK.

### Engineering Hexose Transporter Gal2 for Improving Xylose Assimilating and Carotenoid Production

**FIGURE 4:**
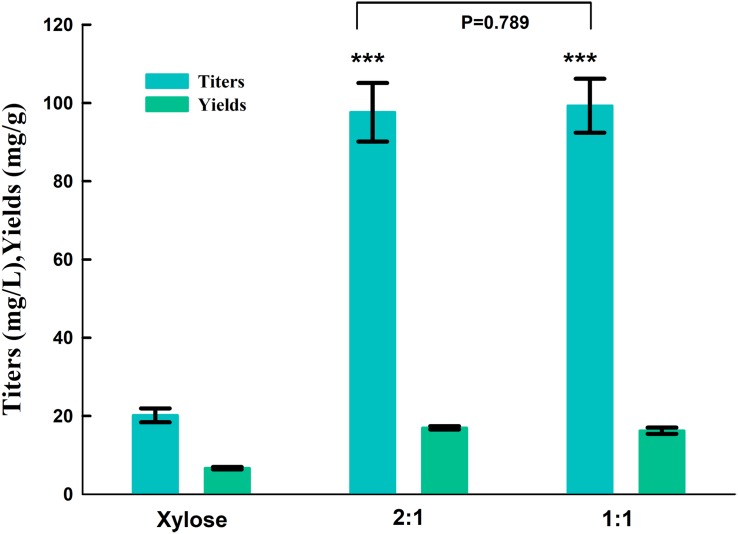
Product titers and yields of strain SC105 from different ration of xylose and glucose in flasks fermentation. Xylose: xylose as sole carbon source at 40 g L^–1^; 1:1: 20 g L^–1^ xylose: 20 g L^–1^ glucose; 2:1, 26 g L^–1^ xylose: 13 g L^–1^ glucose. Each value represents the average ± SD of three biological replicates. Statistically significant differences are denoted ***P < 0.001 (two-tailed Student’s *t*-test).

**FIGURE 5:**
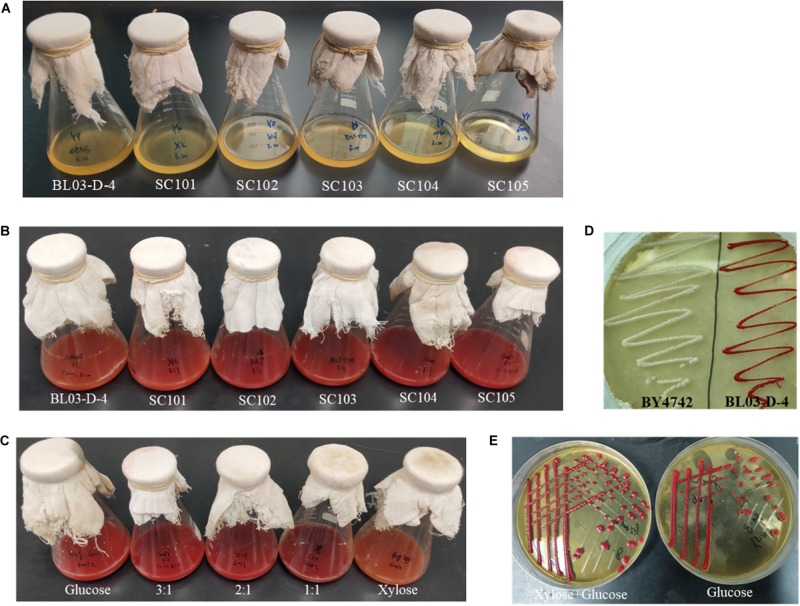
Colors of different carotenoid-producing *S. cerevisiae* mutants. **(A)** Strains were cultured in YP medium without sugars at 30°C for 120 h. **(B)** Strains were cultured in modified YP medium with 30 g L^–1^ xylose and 10 g L^–1^ glucose at 30°C for 96 h; **(C)** SC105 was grown for 96 h on YP medium supplemented with different ration of sugars. Glucose, 40 g L^–1^ glucose; 3:1, 30 g L^–1^ xylose and 10 g L^–1^ glucose; 2:1, 26 g L^–1^ xylose and 13 g L^–1^ glucose; 1:1, 20 g L^–1^ xylose and 20 g L^–1^ glucose; xylose, 40 g L^–1^ xylose. **(D)** BY4741 and BL03-D-4 was grown on YPD plates. After 4 days of incubation at 30°C, the plates were photographed. **(E)** SC105 was grown for 96 h on YP plates supplemented with different sugars. xylose + glucose, 30 g L^–1^ xylose and 10 g L^–1^ glucose; Glucose, 40 g L^–1^ glucose.

Strain SC104 was engineered to carotenoid biosynthesis from xylose-glucose, but xylose assimilating rate was very slow and might be a limitation for efficient carotenoid production. There were no significant improvements of intracellular xylose conversion through overexpression of PK pathway or *Pho13* deletion. Thus, transport might be a bottleneck, especially in the xylose-glucose mixtures. In previous studies, engineering transports were used to improve engineered strains further (Patino et al., 2019). To facilitate xylose metabolism, the mutant hexose transporter Gal2 (N376F, not inhibited by glucose) (Farwick et al., 2014) was overexpressed with the promoter replacement with *SSA1* in strain SC104 to obtain SC105. The subsequent strain SC105 exerted an increasing cell growth and xylose assimilating. About 20 g L^–1^ of xylose was consumed, which was significantly improved compared with SC104 (Figure 2F). Carotenoid titers of SC105 from mixed sugars reached 117.5 mg L^–1^ which was 1.6-fold of that from glucose (Figure 3). The carotenoid yield from mixed sugar was 20.2 mg g^–1^ DCW, which was 1.5-fold higher than that from glucose (13.2 mg g^–1^ DCW). Meanwhile, we found that SC105 produced much lower carotenoid (20.2 mg L^–1^) when xylose was used as the sole carbon source than using mixed sugars (Figure 4). This result was different from that obtained in a previous work where the production yield from xylose shown remarkable improvement than that from glucose (Kwak et al., 2017; Lane et al., 2020). We also found that strains almost could not grow on YP medium without sugars (Figure 5A). Furthermore, the ration of xylose and glucose had no obvious effect on the production of carotenoid in flask fermentation (Figure 5). These results were probably due to that glucose was exhausted quickly in the flask fermentation and there was insufficient cofactor for carotenoid synthesis subsequently (Su et al., 2015). This result also clearly indicated that the addition of glucose had an obvious effect on promoting xylose assimilation and this was similar with our previous work that *E. coli* was engineered for xylitol production from xylose-glucose mixtures (Su et al., 2015). Although our yield was far from the theoretical mass yields (25%, on consumed sugar), SC105 could be used as a starting strain for further systemic engineering (Rude and Schirmer, 2009).

### Fed-Batch Fermentation in a 5-L Bioreactor

**FIGURE 6:**
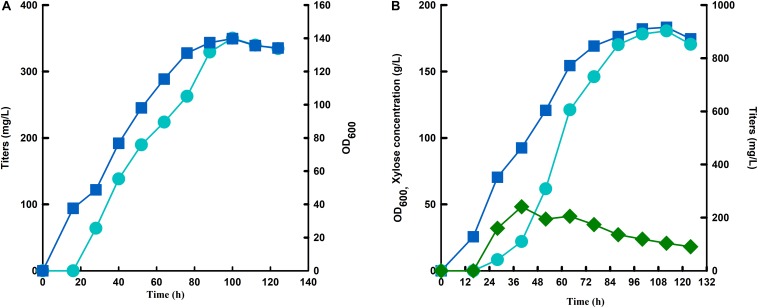
Fermentation of strain SC106 under glucose **(A)** and xylose-glucose mixtures **(B)** in a 5-L fermenter. Symbols: circles, titers; square, OD_600_; diamond, xylose concentration.

Since strain SC105 with mutant hexose transporter Gal2 showed much higher carotenoid contents from mixed sugars than that from glucose or xylose (Figure 5). We speculate that continuous feeding of glucose may facilitate xylose utilization for carotenoid production. As a proof of concept, a fed-batch fermentation with continuous feeding of mixed sugars was performed to increase the production of carotenoid. Strain SC106 recovered with modules of His3-Leu2-Ura3 was inoculated with 10% and cultured with YPD medium. When the initial glucose was consumed, xylose concentration was maintained below 50 g L^–1^ through continuous feedings of mixed sugar (600 g total sugars, xylose/glucose = 3:1). A supplementary fed-batch medium (100 g yeast extract with 10 g L^–1^ KH_2_PO_4_, 2.5 g L^–1^ MgSO_4_, 3.5g L^–1^ K_2_SO_4_, and 0.25 g L^–1^ Na_2_SO_4_) was added together with mixed sugars through the fermentation. Strain SC106 could grow rapidly even though xylose was accumulated at high concentrations in the medium. Finally, in fed-batch fermentation with serial feeding of mixed sugar, the cell density of the engineered strain reached to 183, and 903 mg L^–1^ of carotenoid was produced, which was 2.6-fold of that from glucose fermentation (Figure 6).

## Discussion

Lignocellulosic chemicals are economically feasible target because lignocellulosic biomass is renewable, inexpensive, and abundant. As one of the mostly used host in industrial biotechnology, *S. cerevisiae* has an incomparable capacity to transform glucose from lignocellulosic sugars to bioethanol. Nevertheless, wild-type *S. cerevisiae* cannot assimilate xylose, the second most abundant sugar in lignocellulosic biomass. To unveil the potential advantages of utilizing mixed sugars over glucose by engineered *S. cerevisiae* for carotenoid production, lycopene was chosen as an example of heterologous carotenoid. After several rounds of genetic modification in previous lycopene-producing *S. cerevisiae* BL03-D-4, we have developed a platform capable of assimilating xylose-glucose mixtures for carotenoid production.

Natural xylose-utilizing yeasts can biosynthesize xylulose from xylose through the oxidoreductase pathway which is comprised of xylose reductase (XR) and xylulose dehydrogenase (XDH) (Kwak et al., 2019). Mostly native XRs prefer NADPH as cofactor, but XDH uses only NAD. Thus, a bottleneck in the XR/XDH pathway was generated by the cofactor imbalance between the NADPH-preferred XR and the NAD-dependent XDH. Subsequently, xylitol accumulation and lower production yields was generated (Su et al., 2013). Thus, a mutant XYL1m which was NADH preferred was used for increasing xylose-based carotenoid production in this study and a 25% improvement was obtained. However, *Pho13* deletion slightly increased the cell growth and xylose consumption in our strain. This result might be ascribed to the fact that the positive effect of *Pho13* deletion only appeared when gene expression responsible for xylulose synthesis was sufficiently high (Lee et al., 2014). Since constitutive promoter *TDH*_3_ was employed for overexpression of *XK*, xylose consumption was increased significantly through *Pho13* deletion (Ni et al., 2007; Van Vleet et al., 2008; Bamba et al., 2016). However, our observation was consistent with the previous report that *Pho13* deletion had no significant effect on xylose consumption in strains with the expression of xylose isomerase under the control of *TEF*_1_ promoter (Shen et al., 2012).

Apart from above engineering strategies, engineering xylose transporters were carried out in *S. cerevisiae* to restore the low capacity of endogenous hexose transporters (Li et al., 2016; Wang et al., 2016). Xylose-specific transporter with highly active was much-needed for economically feasible production of chemicals from lignocellulosic sugars (Kim et al., 2013a). However, most sugar transporters of *S. cerevisiae* have much higher specificity toward glucose than xylose (Hou et al., 2017). Xylose uptake might be one of the limited steps in the pathway for xylose assimilation by *S. cerevisiae* in the presence of glucose. For simultaneous utilization of xylose-glucose, an engineered transporter that was xylose specific and not inhibited by glucose was highly desirable. Previous studies have been exactly dedicated to eliminating glucose inhibition in hexose transporters while retaining their xylose transportation capacity (Farwick et al., 2014; Li et al., 2016; Wang et al., 2016). A mutant hexose transporter without glucose inhibition was overexpressed in our strain and the corresponding strain SC105 exhibited 26 and 23% improvement on cell growth and xylose consumption, respectively. And a 42% improvement on carotenoid production was obtained.

There were few studies on developing platform of yeast for carotenoids production from xylose except that native xylose-utilizing *Phaffia rhodozyma* and engineered *S. cerevisiae* (Rodriguez-Saiz et al., 2010; Sun et al., 2012). *P. rhodozyma* was intensively studied for biosynthesis of carotenoid from xylose which convert sugars into a wide variety of carotenoids despite limited consumption rate and growth on xylose. Meanwhile, *S. cerevisiae* has been engineered to synthesize zeaxanthin using xylan from birchwood. Previous work has demonstrated that xylose assimilating in engineered *S. cerevisiae* exerted improvement of isoprenoid production and highlighted great advantages of using xylose as sole carbon source for isoprenoid production (Kwak et al., 2017). However, it would be a complicated situation for mixed sugar, because lignocellulosic biomass was mainly composed of glucose and xylose. Although the carotenoid yield obtained in our study was far from the values of previous work (Chen et al., 2016; Ma et al., 2019; Shi et al., 2019). High carotenoid yield was not the main purpose of this study, because a basic strain with a relatively low carotenoid production (with not many genetic modifications) was chose as the starting strain. Our main goal was to develop a platform that could efficiently utilize xylose-glucose mixtures for carotenoid production.

## Data Availability Statement

The datasets analyzed in this manuscript are not publicly available. Requests to access the datasets should be directed to zhuhh@gdim.cn.

## Author Contributions

HZ conceived the project. BS designed the experiments, wrote and revised the manuscript. BS and DS performed the experiments.

## Conflict of Interest

The authors declare that the research was conducted in the absence of any commercial or financial relationships that could be construed as a potential conflict of interest.

## Supplementary Material

DATA SHEET S1 Table S1 and Figure S1.

DATA SHEET S2 Raw Data.
