# Supplementary material for: Metabolic Engineering of Saccharomyces cerevisiae for Enhanced Carotenoid Production From Xylose-Glucose Mixtures
Source: Front Bioeng Biotechnol. 2020 May 14;8:435. doi: 10.3389/fbioe.2020.00435 (PMC7240070; doi:10.3389/fbioe.2020.00435)

Supplementary Material

# Supplementary Tables

**Table S1.** Primers used in this study. Homologous overhang-nucleotides (underlined); genomic target (red, bold); short synthetic terminator (blue, italics); point mutation (red, capital).

| Primers | Sequence (5’-3’) |  |
| --- | --- | --- |
| gRNA-UP-F  gRNA-UP-R  gRNA-XK-F  gRNA-R  EFT1-F-2  EFT1-F  EFT1-R  XYL1-F  XYL1-R  XYL1-R-2  PGK-F  PGK-R  XYL2-F  XYL2-R  XYL2-R-2  HSP82-F  HSP82-R  HSP82-R-2  pACYC-XK-F  pACYC-XK-R  EFT1-XYL1-F  EFT1-XYL1-R  PGK-XYL2-F  PGK-XYL2-R  XK-check-F  XK-check-R  720-F  HSP104-F  HSP104-R  xpk-F  xpk-R  xpk-R-2  SSA1-F0220  SSA1-R0220  pta-F  pta-R  pta-R-2  720-R  gRNA-Pho13-F  Pho13-UP-F  Pho13-UP-R  Pho13-DOWN-F  Pho13-DOWN-R  Pho13-check-F  Pho13-check-R  gRNA-Gal2-P1  SSA1-F0523-2  SSA1-F0523  SSA1-R  Gal2-F  Gal2-R  Gal2-R-2  Gal2-check-F  Gal2-check-R  911-F  His3-F  Ura3-R  911-R | Primers for constructing strains SC101 and SC102  ctagagatctgtttagcttgcctcgt  gatcatttatctttcactgcggagaagtttc  cttctccgcagtgaaagataaatgatc**gacgcagggaatagccgctg**gttttagagcagaaac  cattttgaagctatgggatctgacattattattgttggaag  gcaagctcaaaagagatatatatatcatccagcatagactactattaatt  atcatccagcatagactactattaattagatagagtatgaaagaaagaagctgacg  ttttatctgttattaaaaattcttgggtgc  gcacccaagaatttttaataacagataaaaatggctactctattaaaattgaacaatg  *tttgaaaaaatttatttctagacagttatata*ctaggcaaatatagggatcttgtccca  taacatcaattcaagaaggagggaatttgaaaaaatttatttctagacagttat  ttccctccttcttgaattgatgtta  tgttttatatttgttgtaaaaagtagataattac  tatctactttttacaacaaatataaaacaatgtccgataacccaagtgttattctta  *tttgaaagatactctttatttctagacagttatata*ttactctgggccgtcaatgata  aggaataaaaagtcccgcgccactaatttgaaagatactctttatttctagaca  ttagtggcgcgggactttttattcct  tctgtctctgaattactgaacacaacatatctttgcgtgtttgtttgtgcttttg  aagacattgtgttggaaacctctcttgtctgtctctgaattactgaacacaacat  Primers for constructing plasmid pACYC-XK  gagaggtttccaacacaatgtcttaataactagcataaccccttggg  tatatatatctcttttgagcttgcacttcaggtgctacatttgaagag  Primers for mutation of XYL1  gcaagctcaaaagagatatatatatcatccagcatagactactattaatt  tctggtttactggaGttgggaatgat  tatcattcccaaCtccagtaaaccaga  aagacattgtgttggaaacctctcttgtctgtctctgaattactgaacacaacat  atgtcctagacccttaataagcaaaacactc  acccttctttgtgtgataatgcggaaga  Primers for constructing strain SC103  gtgtgcgaaaagtactttggatcagcctttccttcacgttcggtccactt  agcctttccttcacgttcggtccacttcgattcaaaggcgttattcagcatcat  atattctgtatattttatggtacgtgtagttga  actacacgtaccataaaatatacagaatatatgaatatcgattcaacagattacctga  *tttgaaagatactctttatttctagacagttatata*tcacttaagtggttgccagcg  aacaatttgtcgacaaccgagcctttgtttgaaagatactctttatttctagacag  ctgtctagaaataaagagtatctttcaaacaaaggctcggttgtcgacaaattgtt  attatctgttatttacttgaatttttgtttcttgtaatac  acaaaaattcaagtaaataacagataatatggaacttatgaaaaaaatatgggaagct  *tttgaaagatgatactctttattaaatatata*ttatttaatcccttgtgcttgaacag  cctagcttaggctaagaaactccttctttgaaagatgatactctttattaaatatata  ggatagataatgggggcgcgcctgcctagcttaggctaagaaactccttc  Primers for constructing strain SC104 with the deletion of Pho13  cttctccgcagtgaaagataaatgatc**ggttatacatttcccggtgc**gttttagagc  actgctgatggagcaatatttcaaatc  ccattcttccccggagtctgcttgttataaaccgattgcgtca  tttataacaagcagactccggggaagaatggattatccggag  tcatcaatacatccgtaacccgcccgaa  aattgccaagattcagacgccgatcgca  aaacctatcataagacatgaagggcaaa  Primers for constructing strain SC105 with the mutation N376F of Gal2  cttctccgcagtgaaagataaatgatc**ttggacgatattaaggcaga**gttttagagc  ctaatccaaggaggtttacggaccaggggaactttccagattcagatcacagcaa  ctttccagattcagatcacagcaacaaaggctcggttgtcgacaaattgtta  attatctgttatttacttgaatttttgtttcttgtaatac  caaaaattcaagtaaataacagataatatggcagttgaggagaacaatatgcct  aactaaagaaagtggaggcaaagAAgactacaccaat  tacgatgtcccaagttttcgacagtccacaaactaaagaaagtggaggcaaagAAg  gcttccaagacgacagtaatatgtctc  aggcaactggcgcccaggttgtggca  actttccagaaacagatatctatattttataacaaagacgtacggcatcatttaaagt caaagacgtacggcatcatttaaagtctattactcttggcctcctctagtaca  taaagtatgtatcgggaagtctccacctactgttacttggttctggcgaggtat  cagactcagaaaattttatgcaacaacattaaagtatgtatcgggaagtctccacc |  |

Figure S1. HPLC analysis of the standard (A) and carotenoid extract of BL03-D-4 (B). The spectrum of peak in standard (C) and carotenoid extract of BL03-D-4 (D). (E) Spectral scanning for the carotenoid extract of BL03-D-4 by using UV-spectrophotometer.


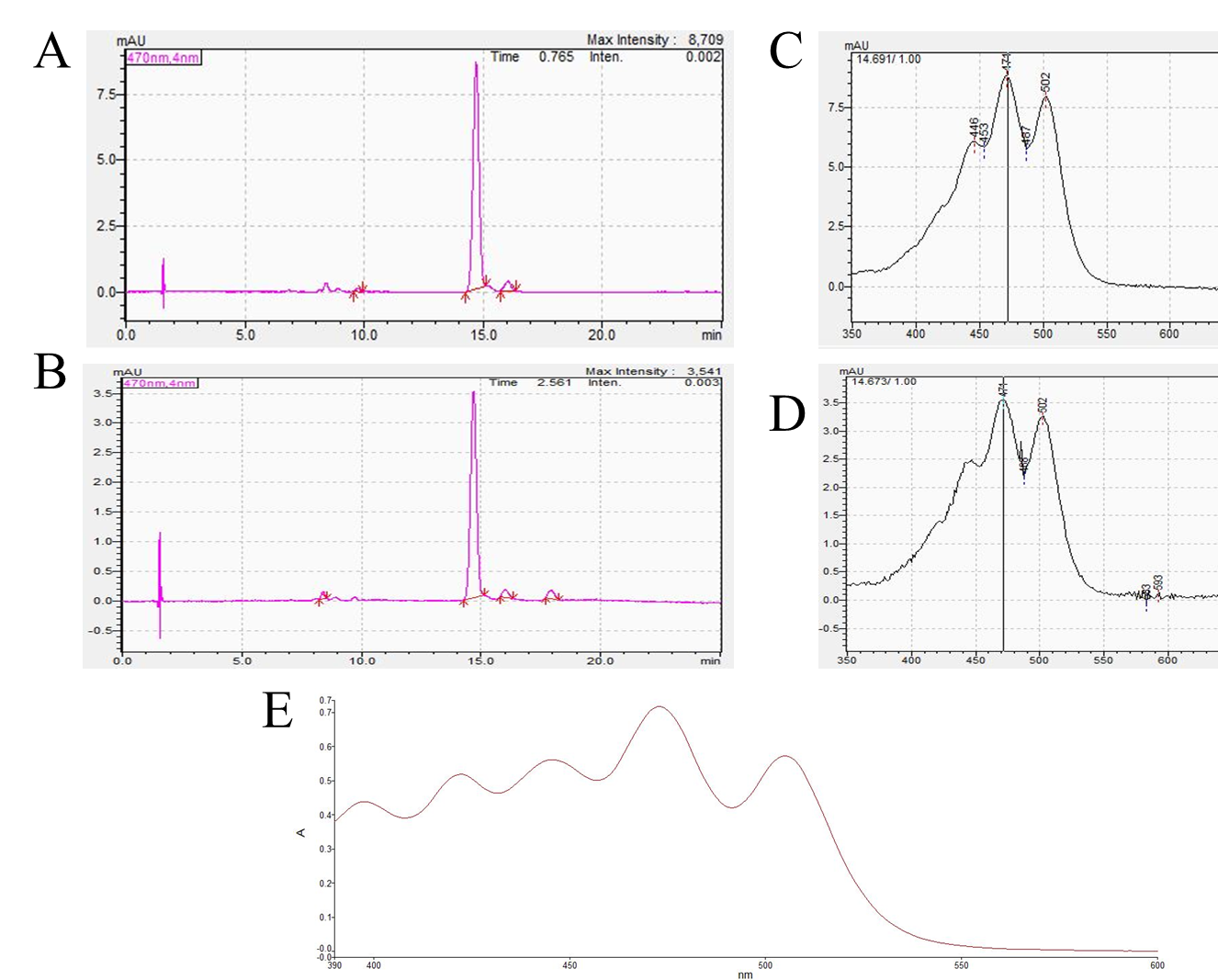

Supplement: DATA SHEET S1 — Table S1 and Figure S1. [file Data_Sheet_1.docx]
